# P69 A cross-sectional audit of antibiotic utilization and AWaRe stewardship alignment in hospitalized patients

**DOI:** 10.1093/jacamr/dlag102.075

**Published:** 2026-06-26

**Authors:** S Harshitha, Afrin Siddique, Angel Jennifer, D Suresh Kumar

**Affiliations:** SRIHER, Chennai, Tamil Nadu, India; MMM Hospital, Chennai, Tamil Nadu, India; MMM Hospital, Chennai, Tamil Nadu, India; MMM Hospital, Chennai, Tamil Nadu, India; Best Of IDs, Chennai, India

## Abstract

**Background:**

Antibiotic utilization audits are well established in high-income settings but remain limited in developing healthcare systems, where the burden of antimicrobial resistance is high and stewardship practices are still evolving. Prescribing is often empirical, with variable adherence to evidence-based principles. The WHO AWaRe (Access, Watch, Reserve) classification provides a structured framework to evaluate antibiotic selection; however, its application in routine inpatient audits is still emerging.

**Objectives:**

This study aimed to evaluate antibiotic utilization, AWaRe distribution, duration of therapy, and stewardship alignment in hospitalized patients using an AWaRe-based audit approach.

**Methods:**

A retrospective cross-sectional audit was conducted in a tertiary care hospital in South India, including 257 hospitalized patients; outpatients were excluded. Data collection was performed during March 2026 using a structured tool developed and validated by the antimicrobial stewardship team and recorded via Google Forms. Data were collected by trained physician associates and clinical pharmacists under the supervision of an Infectious Diseases specialist, ensuring consistency and accuracy. Variables included antibiotic use, duration of therapy, AWaRe classification, microbiological evidence, and Infectious Diseases consultation. Stewardship alignment was assessed using predefined proxies: microbiological confirmation for definitive therapy, culture submission for empirical therapy, and ≤2 days duration for prophylaxis. Statistical analysis included descriptive statistics, independent t-tests for comparison of antibiotic duration, and multivariable linear regression to identify factors associated with duration of therapy.

**Results:**

Antibiotics were prescribed in 44.4% (114/257) of patients. Prescriptions were predominantly from the Watch group (72.8%), followed by Access (10.5%) and Reserve (8.8%). The mean duration of therapy was 3.62 days (median 3; IQR 2–5). Approximately 60% of prescriptions met stewardship alignment criteria. Cultures were obtained in 67.3% of cases, with a positivity rate of 28.1%. Patients who underwent culture testing had significantly longer antibiotic durations (t-test, *P*=0.006), while Infectious Diseases consultation was not significantly associated with duration (*P*=0.735). Multivariable linear regression (R²=0.21) identified empirical therapy and absence of culture as predictors of shorter duration, and lack of ID consultation as a predictor of longer therapy.

**Conclusions:**

In this audit, 44.4% of patients received antibiotics, with predominant Watch group use (72.8%) and limited Access use (10.5%) despite a culture positivity of 28.1%, indicating suboptimal alignment with microbiological evidence. The retrospective single-centre design is a limitation. Regular AWaRe-based audits can help guide targeted stewardship and improve antibiotic use in routine practice.

**Table 1.** Antibiotic use and stewardship indicators

Parameter	Value
Total patients, *n*	257
Antibiotic use, % (*n*)	44.4 (114)
Watch group use, %	72.8
Access group use, %	10.5
Reserve group use, %	8.8
Duration, mean, days	3.62
Stewardship-aligned prescriptions, %	∼60
Cultures obtained, %	67.3
Culture positivity, %	28.1

